# Health Impact Assessment of Artisanal and Small-Scale Gold Mining Area in Myanmar, Mandalay Region: Preliminary Research

**DOI:** 10.3390/ijerph17186757

**Published:** 2020-09-16

**Authors:** Win Thiri Kyaw, Xiaoxu Kuang, Masayuki Sakakibara

**Affiliations:** 1 Research Institute for Humanity and Nature, 457-4 Motoyama, Kamigamo, Kita-ku, Kyoto 603-8047, Japan; xxkuang@chikyu.ac.jp (X.K.); sakaki@chikyu.ac.jp (M.S.); 2 Graduate School of Science & Engineering, Ehime University, 2-5 Bunkyo-cho, Matsuyama City, Ehime Prefecture 790-8577, Japan; 3 Faculty of Collaborative Regional Innovation, Ehime University, 3 Bunkyo-cho, Matsuyama City, Ehime Prefecture 790-8577, Japan

**Keywords:** artisanal and small-scale gold mining, health, respiratory function, mercury intoxication, Myanmar, spirometry

## Abstract

Increasing artisanal and small-scale gold mining (ASGM) in developing countries has raised health concerns in mining communities. A preliminary health survey was conducted in Thabeikkyin Township, Mandalay Region, Myanmar, in February 2020 to assess the health conditions of an ASGM community. Respiratory function and other clinical assessments were evaluated in miners and non-miners, and participants’ hair was analyzed for heavy metals. Respiratory function of miners was similar to that of non-miners. However, miners’ respiratory function declined with longer mining activity duration. In total, 3 out of 18 miners showed neurological signs and symptoms of chronic mercury intoxication. The median concentration of the hair mercury was significantly higher in miners than non-miners (*P* = 0.01), and 9 out of 18 miners and 2 out of 11 non-miners showed the warning level of mercury. We found that, despite an association between declining respiratory function and length of time mining, only a minority of miners showed clinical features of chronic mercury intoxication. Further clinical surveys with a larger sample size are necessary to determine the broader health status of this community. In addition, clinical indicators such as pulmonary function tests are recommended as additional criteria for the diagnosis of mercury intoxication.

## 1. Introduction

There are approximately 15 million miners working in the artisanal and small-scale gold mining (ASGM) sector, and approximately 100 million people globally residing in ASGM communities [1]. As ASGM activity increases, concern also increases regarding the miners’ health. In addition, the ASGM sector uses the largest amount of mercury (Hg) globally and is responsible for emitting the largest anthropogenic source of Hg into the environment [2]. Hg exists in three forms: elemental Hg (liquid Hg), inorganic Hg, and organic Hg or methylmercury (MeHg). Elemental Hg is used in the ASGM process for binding gold in the ore, known as gold-Hg amalgam, which is further smelted to retrieve the gold [2]. Smelting the amalgam releases Hg into the atmosphere, accounting for the environmental pollution of ASGM communities. The majority of ASGM miners are exposed to Hg from inhaling the vaporized form of Hg released during smelting of the amalgam [3], and it is readily absorbed through the lungs [4]. The vaporized form of Hg emitted into the atmosphere eventually deposits in the soil and the sediment of lakes, rivers, bays, and oceans, and is transformed by anaerobic organisms into MeHg. As MeHg is diffused into the water column, it is absorbed by phytoplankton, which are ingested by zooplankton and fish; this results in food chain contamination. It becomes concentrated over time, especially in long-lived predatory species such as sharks and swordfish [5]. Therefore, members of the ASGM community can also be exposed to MeHg from food sources regardless of their occupation as a miner, or not.

A number of studies worldwide have reported that Hg released from ASGM activity impacts the health of ASGM miners [6,7,8,9,10,11,12,13,14,15]. The pulmonary and renal systems are harmed by the intrusion of Hg vapor, but the nervous system can also be impaired. Occupational lung diseases, such as asbestosis, malignant disease, mesothelioma, silicosis, lung cancer, tuberculosis, and pneumoconiosis, are associated with mining activity [16]. Therefore, some studies have included a pulmonary function test both to evaluate the respiratory health of ASGM miners [17], and to monitor the impact of Hg on the health of ASGM miners over time as an early biomonitoring tool [18].

Moreover, as Hg vapors emitted from ASGM activity travel by wind to neighboring communities, people without occupational exposure to Hg can also experience its harmful effects. A comprehensive review reported that not only ASGM miners but also residents living downstream of mining activities commonly experience nervous system impairment (i.e., tremor, ataxia, memory problems, and vision disorders) owing to the exposure of Hg released from ASGM activities [4]. Members of ASGM communities face adverse effects to their nervous, renal, and autoimmune systems from Hg exposure [4].

Despite these harmful health effects, ASGM is practiced in developing countries as a low-income driven livelihood choice in rural areas because of the advantages it offers; namely, little requirement for investments, technological skill, or education. Myanmar is a developing country where gold mining activities are prevalent, particularly in the Mandalay Region, Sagaing Region, Bago Region, and Kachin State [19]. Previous reports show that the environment of Myanmar is polluted by Hg released from ASGM activities [19] and that the groundwater and atmosphere in ASGM areas are also contaminated by Hg [20].

To date, only a few reports have been published on the health impact in ASGM communities in Myanmar; however, these studies do not offer detailed clinical assessments of the ASGM community [21]. Thus, health information regarding the adverse effects of ASGM mining on the communities within mining areas is insufficient. This preliminary health survey aimed to detect the extent of the health impact among ASGM miners and their family members in the Thabeikkyin Township, Mandalay Region, by evaluating pulmonary function tests and other clinical evaluations, including Hg and other heavy metals in the hair. It is the first report to offer a clinical assessment of the ASGM community in Myanmar. Findings from this survey can help to establish the parameters of future health surveys in ASGM communities. This article discusses the nature of the ASGM practices in the study area, clinical evaluation of Hg intoxication focusing on neurologic and respiratory systems using pulmonary function test and the analysis of Hg and other heavy metals contents of participants’ hair, and the correlation of these findings to the health status of the focal ASGM mining community.

## 2. Materials and Methods

A preliminary health survey was performed among ASGM miners and their family members in Chaung Gyi (also spelled as Chaunggyi) Village, Chaung Gyi Village Tract, Thabeikkyin Township, Pyinoolwin District, Mandalay Region, Myanmar, in February 2020. In total, 29 participants were randomly recruited from the residents of Chaung Gyi Village (18 men and 11 women). The exclusion criterion was the presence of moderate to severe systemic illness. The individual responsible for the survey provided a clear explanation of the purpose, method, merit, and demerit of the preliminary survey, and also received a signed informed consent from the participants before the survey. All participants underwent a general interview, a general health assessment, a neurological system assessment, a respiratory function assessment using a spirometer (Spirodoc; Medical International Research, Rome, Italy), and hair sampling for the analysis of Hg and other heavy metals. The participants were then divided into two groups; miners represented individuals currently engaged in mining activities, and non-miners represented individuals not currently engaged in mining activities. In the non-miner group, nine individuals were previously involved in ASGM occasionally but had stopped working as a miner at least 10 years before. The health questionnaire for this survey was approved by the ethical committee of the Research Institute for Humanity and Nature (RIHN).

### 2.1. Study Area

**Figure 1:**
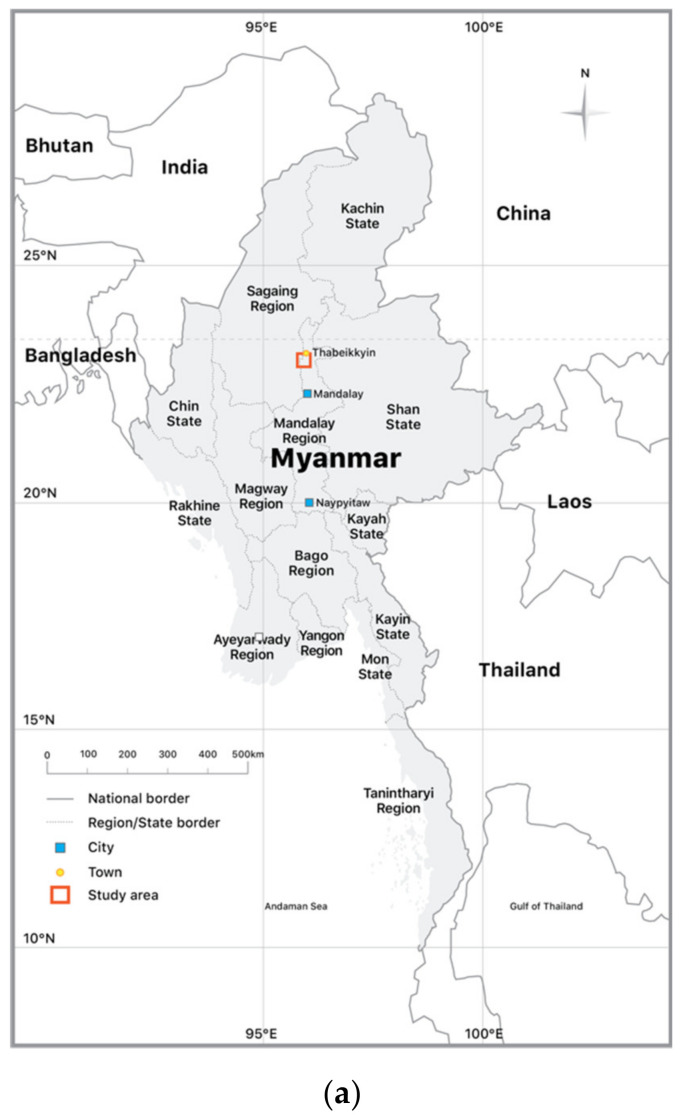

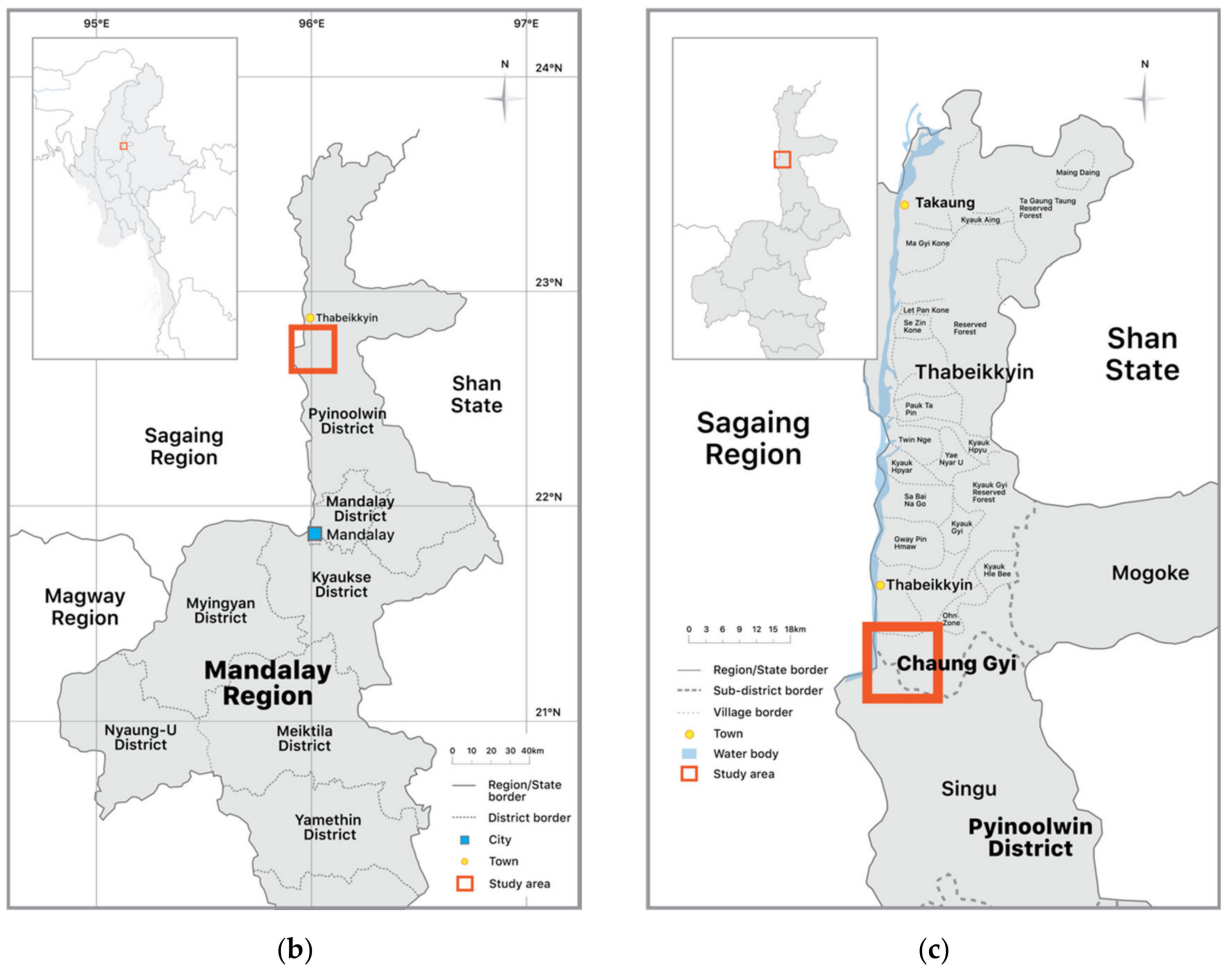
Study area. (**a**) Map of Myanmar with states and regions. (**b**) Map of the Mandalay Region with districts. (**c**) Chaung Gyi Village, Thabeikkyin Township, Pyinoolwin District, Mandalay Region.

Thabeikkyin Township (Figure 1) is mainly composed of two village tracts: Chaung Gyi and Ohn Zone. This study was conducted only in Chaung Gyi Village of the Chaung Gyi Village Tract as a preliminary health survey owing to limitations in the duration of the study. According to March 2019 data, Chaung Gyi Village has 1772 households and a population of 8375 people. Gold mining is the primary livelihood in Thabeikkyin Township. The mining sector is composed of both large-scale and small-scale mining. The large-scale mining sector includes mining businesses operated by mining companies that receive permits issued by the National Mining Enterprise No. 2, which is regulated by Myanmar’s Ministry of Natural Resources and Environmental Conservation. There are 54 licensed mining companies in Thabeikkyin Township. In contrast, ASGM in Thabeikkyin Township can be either formal or informal and are operated by individuals or as a family business.

In Thabeikkyin Township, the ASGM process is as follows: gold ore is mined underground, approximately 20–30 m below the surface, by digging at a level after making a vertical tunnel. The gold ore is then prepared for drying and is crushed into a powder using a machine. The gold particles are separated from the powder by gravity separation. Hg is added to the pan to form a gold-Hg amalgam, which is later squeezed in a cloth to drain the liquid Hg. As the final step, the amalgam is burned to refine the gold, and Hg vapor is emitted into the atmosphere [20]. Some of the refining processes in the Chaung Gyi Village take place in the same compound where people live. Vapors from smelting are emitted into the atmosphere of the whole village, causing concern for environmental Hg pollution.

### 2.2. General Interview

A general interview was conducted by one medical doctor and trained staff directly with the survey participants in the local language. The general interview included data (e.g., name, birth date, sex, address, occupation, and education) and a questionnaire regarding exposure to Hg and other heavy metals (e.g., how far they live from the mining area, duration of occupation as a miner, type of mining activities, history of handling Hg, use of personal protective equipment such as gloves and masks while handling Hg, and the participant’s family history of occupation). The question regarding the type of mining activities was a closed question with preset options: digging and crushing the ore, panning, amalgamation, or refining gold by smelting.

### 2.3. General Health Assessment

A medical doctor conducted the medical history and physical examination of the participants. The participants were asked about present health concerns, past medical and surgery history, smoking status, and symptoms of acute and chronic Hg intoxication (e.g., metallic taste, excessive salivation, tremors, sleep disturbances, tiring easily, feeling sleepy or drowsy, lack of energy, feeling weak, problems concentrating, forgetfulness, being nervous, being sad, heart palpitations, headaches, nausea, appetite loss, weight loss, hair loss, numbness and prickling, and aching (perioral dysesthesia, stocking-glove pattern)). Participants who had been smoking for 10 years at the time of the survey were classified as smokers, and those who had quit smoking 10 years before were classified as nonsmokers. The interviewer asked the participants questions regarding the number of cigarettes they smoke per day and the duration of smoking. The Brinkman index was applied to represent the grading of smoking, which was calculated by multiplying the number of cigarettes smoked per day with the duration of smoking. Weight, height, blood pressure, pulse rate, and respiratory rate were evaluated during the physical examination.

### 2.4. Neurological System Assessment

A neurological system assessment was conducted to examine the mouth, teeth, and eye conditions in relation to Hg intoxication and the visual field; sensory testing such as touch, pinprick, vibration, and temperature; touch test using a monofilament tester (Semme Weinstein Monofilament; SAKAImed, Tokyo, Japan) and a quantitative manual gauge (Yufu Seiki Co., Ltd., Tokyo, Japan) in participants with an impaired sensory test; motor test for muscle power and reflex; ataxia of gait; tremor assessment, including finger-to-nose test, dysdiadochokinesis, and Romberg’s test.

### 2.5. Respiratory Function Assessment Using a Spirometer

A complete respiratory system assessment and spirometry test were performed by the same medical doctor using a portable spirometer (Spirodoc). The spirometer was connected to a laptop on which the spirometer software was installed, and participants were requested to perform the test. Before performing the spirometry test, the medical doctor explained the instructions to every participant and offered a mock performance for demonstration. A disposable turbine with cardboard mouthpiece (FlowMIR; Medical International Research) was attached to the spirometer, and participants were asked to blow air into the turbine while wearing a nose clip. The turbines were disposed and replaced after each use and before the next spirometry test.

Spirometry automatically calculates the results of respiratory function relative to the age, sex, height, and weight of the test taker. Spirometry evaluates the measured value, prediction value, and percentage of prediction value of forced vital capacity (FVC), the forced expiratory volume in 1 s (FEV1), FEV1/FVC%, and spirometry interpretation. Because “Myanmar” was not an option for selection under ethnicity, “Oriental” was selected. The test was repeated three times for all participants, and the best performance was selected by the spirometer software. In the case of an unsuccessful test performance, participants rested before repeating the test until a successful reading was achieved.

### 2.6. Analysis of Hg and Other Heavy Metals in the Hair

Hair samples were washed with a neutral detergent, distilled water, and acetone, and then dried in an oven at 60 °C for 4 h. A 100 mg sample was accurately weighed into a Teflon container. Then, 1 mL of HNO_3_ (Ultrapure analytical reagent; Tama Chemical Co., Ltd., Kawasaki City, Japan) was added to the samples before heating on a hot plate at 200 °C for 1 h. The sample was diluted with ultrapure water. The total Hg in the hair was analyzed using a reducing-vaporization Hg analyzer (RA-43000; Nippon Instruments Co., Ltd., Tokyo, Japan) according to the Hg analysis manual (Ministry of the Environment, Tokyo, Japan). Lead (Pb), arsenic, and other heavy metals were analyzed using inductively coupled plasma mass spectrometry (7500cx, Agilent Technologies, Inc., Wilmington, DE, USA). The analysis of each sample was made three times to ensure the reliability of the data.

### 2.7. Statistical Analysis

The values of the variables were presented as median (interquartile range), as some of the data were not normally distributed. FVC and FEV1 were evaluated as the main variables and were compared between the miner and non-miner groups. In the first stage, a Chi-square test of independence was performed to examine the relation between smoking and the spirometry results of the miner and non-miner groups. Then, a comparison was made between the miner and non-miner groups for FVC, FEV1, Hg, and Pb level in scalp hair. We also examined the correlation among FVC, FEV1, Hg, Pb, age, smoking status, and duration of mining between both groups. The Mann–Whitney test was used for comparison, and Spearman’s Rho correlation was used for correlation analyses. The level of significance was 95% with α set at 0.05. 

## 3. Results

### 3.1. Characteristics of the Participants and Results of the General Interview

Participant characteristics are shown in Table 1. The age of non-miners was found to be higher than the miners, particularly because older individuals prefer non-mining jobs or they become dependent with advanced age. Similarly, the mean BMI of non-miners was greater than the miner group. However, the gender composition of the two groups are not different. 

### 3.2. Result of General Interview

Participants lived at the mean distance of 6.8 km (range, 0.03–32 km) away from mining area or gold refining place. ASGM miners who participated in this study worked in three types of scenarios: as a miner in a small group with daily wage earnings, long-term family business operating a gold shop, and short- to long-term mining as a mine owner. Regarding the type of mining activity, most of the miners worked in all stages of the mining process, depending on the necessity of the workload in the mine. Of the miners who participated in this study, 72.2% performed digging and crushing of the ore as their major mining activity; 88.8% indicated that they handled Hg for the amalgamation and smelting processes; 66.6% indicated that they did not wear personal protective equipment (e.g., gloves and masks) while working.

### 3.3. Clinical Findings and Analysis of Heavy Metal

In the neurological assessment, three female participants (16.7%) in the miner group complained of chronic tremors and numbness of their digits. Mild tremors and ataxia were detected in the neurological assessment. These three miners were involved in panning and the amalgamation process continuously for more than 5 years with exposure to Hg, and their symptoms match the symptoms of chronic Hg intoxication.

Hg and other heavy metals are described in Table 2. The median (interquartile range) of the hair Hg level was significantly higher in miners at 0.93 μg/g (interquartile range (IQR), 0.72 to 1.44 µg/g) than in non-miners at 0.63 μg/g (IQR, 0.53 to 0.67 µg/g). The German Human Biomonitoring (HBM) Commission standard (2007) [22] was applied for the determination of safe Hg levels in the hair. According to HBM I and II, a safe Hg level is within the range 1 part per million (ppm) or less, and a high Hg level is considered to be greater than 5 ppm. The alarming Hg level among the miners ranged between HBM I and HBM II [22]. Of the miners, 10 persons, 55.5%, had a safe level of Hg (lowest value, 0.5 μg/g) and 7 persons, 38.8%, had an alarming level of Hg. There was only one participant with a high level of Hg (8.9 μg/g) who worked the process of refining gold, and this outlier caused the large range of Hg levels in the miner group (range, 0.5–8.5 μg/g). Among the non-miners, 9 persons, 81.8%, showed a safe level of Hg and 2 persons, 18.2%, showed an alarming level of Hg. Among the heavy metals analyzed, Hg and Pb levels were considered for interpreting the correlation with the variables. The other heavy metals, such as lead (Pb), arsenic (As), cadmium (Cd), and copper (Cu), in hair samples were within the reference range, and no difference was found between the miner and non-miner groups for these.

### 3.4. Spirometry Test and Analysis of Lung Function

Spirometry test results and other variables are described in Table 3. The smoking grading of all participants was mild according to the Brinkman index (≤199). There was no specific grading of smoking status for Myanmar nationals. The number of smokers and non-smokers in both groups were found to be independent between the miner and non-miner groups. The relationship between these variables was insignificant (χ^2^: [1, *N* = 29] = 2.08, *P* = 0.14), which means that smoking did not skew the spirometry test results between miners and non-miners. 

The parameters of pulmonary function (FVC, % prediction of FVC, FEV1, % prediction of FEV1, and spirometry interpretation) are detailed in Table 3. Among the parameters, FVC and FEV1 were studied for a correlation with other variables and for comparison between the two groups.

As shown in Table 4, the variables were compared between miners and non-miners. The miner group had an increased Hg level in their hair compared with the non-miner group, although the values were lower than those considered to be harmful for human health (*P* = 0.01). There was no significant difference in the pulmonary function test parameters, FVC and FEV1, and Pb level in their hair between the two groups.

Table 5 demonstrates the correlation of variables between miners and non-miners. In both groups, increasing age was associated with decreasing lung function. There was also a correlation between smoking and FVC or FEV1 in both groups, indicating that smoking had some adverse effect on lung function among both miners and non-miners. Both FVC and FEV1 values also declined with increasing duration of mining activity. However, there were no correlation among Hg and Pb levels and lung function.

**Figure 2:**
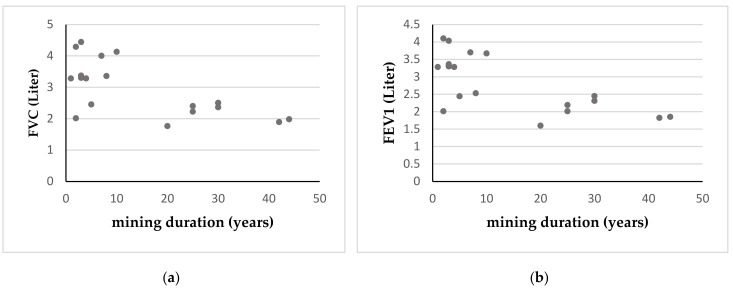
Correlation between the duration of mining, FVC, and FEV1 among miners. (**a**) Correlation between the duration of mining and FVC (*P* = 0.001 by Spearman’s Rho correlation test); (**b**) correlation between the duration of mining and FEV1 (*P* = 0.007 by Spearman’s Rho correlation test).

Figure 2 shows the correlation between the duration of mining, FVC, and FEV1 among miners. Miners with longer duration of mining activity demonstrated decreased FVC and FEV1.

## 4. Discussion

In this study, the clinical assessments of Hg intoxication, respiratory function, and heavy metal contents in the hair of miners and non-miners from an ASGM community were evaluated to determine the community’s health impacts. Tremors, psychological disturbances, erethism, and gingivitis have been identified previously as the major clinical manifestations of chronic Hg poisoning from Hg vapor inhalation [23]. In this study, approximately 16% of the miner group exhibited signs and symptoms of chronic Hg intoxication, such as impairment of the nervous system, whereas non-miners showed no abnormal findings. The respiratory function between the miner and non-miner groups was also not different, although poor results were hypothesized in miners. This lack of difference may be due to the study’s small sample size. However, in the miner group, FVC and FEV1 values were decreased with longer duration of mining activity, indicating that the miners’ respiratory function can be affected chronically. A minor limitation of our study was the absence of reference data on FVC and FEV1 among healthy Myanmar nationals to compare with our results. ASGM miners accidentally inhale elemental Hg emitted during smelting. Upon inhalation, elemental Hg passes through the alveolar membrane, is absorbed into the blood, and travels to the tissues [24]. Therefore, the respiratory system is primarily affected in individuals who have been exposed to elemental Hg. Hg vapor exposure has been reported to cause clinical pulmonary dysfunction in previous case reports [25,26,27]. Inhalation of Hg vapor in high concentration causes fatal acute lung injury, such as acute respiratory distress syndrome [26,27]. Based on this, a respiratory function test is recommended as an evaluation tool to detect early Hg intoxication.

Furthermore, the inhaled elemental Hg vapor in the bloodstream can cross the blood–brain barrier and oxidize into Hg^++^, which further accumulates in the brain [28]. The oxidized form of elemental Hg binds strongly to selenium or sulfhydryl groups (SH) in the brain, which may result in persistent brain deposits [29]. Elemental Hg is also deposited in the kidneys, similar to the brain, for several weeks or months [30]. Therefore, the longer the duration of exposure to elemental Hg, the greater the cumulative damage to the central nervous and renal systems. Accordingly, the urinary concentration of Hg, which is derived directly from kidney tissue, can indicate a state of chronic exposure to elemental Hg and is considered to be the most reliable bioindicator of chronic elemental Hg intoxication [28]. 

In our study, the value of Hg content in 7 out of 18 miners was between 1 µg/g and 5 µg/g, which does not represent a level harmful to human health but demonstrates that the value is in a warning range, according to HBM Commission Standard. Another recent study conducted in our survey area also reported that the average hair Hg content among ASGM miners was lower than a level harmful to the human health, [20] and is consistent with findings of this study. Hair Hg content has been used as a biomarker to estimate chronic exposure to Hg in many ASGM studies because of its simple and noninvasive properties. In areas where ASGM activity takes place, the Hg from the waste disposed during the amalgamation method may enter the underground water and water channel, and is methylated by anaerobic organisms into MeHg, which further infiltrates into the food chain. Prior studies have found up to 80% of the total Hg analyzed in hair among consumers of fish can be attributed to MeHg acquired from diet [31]. Therefore, hair Hg is an excellent biomarker to detect MeHg exposure. Thus, the relatively low level of hair Hg content of the miners of the study area compared to the other ASGM areas across the world indicates less exposure to MeHg, which may be due to variation regarding time span of the use of Hg in ASGM process and the amount of Hg usage. Historically, Hg has been used in the ASGM study area for around 30 years, which is far less than those in other MeHg exposure related recorded areas. The estimation of Hg hair content can detect only minor components of elemental Hg. Therefore, hair Hg is not a robust indicator to determine the occupational exposure to elemental Hg from inhaling the vapor form, as in the process of ASGM mining, unlike urinary Hg, which can represent exposure to elemental Hg [3,32]. 

Pb, As, Cd, and Cu coexist and are associated with gold in ASGM sites due to the ASGM activity. Harmful levels of these heavy metals were undetected in hair samples from both groups. However, according to a recently published study, higher levels of heavy metals including As, Cd, Pb, and Hg compared to the other countries were detected in the soil samples of ASGM sites in Myanmar; especially in the amalgamation process, according to their characteristics of greater density than the other minerals, and the author suggests that miners can suffer from the adverse effects on health from direct exposure to these heavy metals, and also residents can be affected through inhalation and dermal exposure [33]. Exposure to these heavy metals is associated with an increased risk of cancer. It is known that chronic exposure to Cd may affect the kidneys, bones, and lungs [34], and exposure to Pb exerts an adverse effect on the nervous, renal, and cardiovascular systems [35]. Therefore, together with an analysis of these heavy metals, thorough clinical evaluations of heavy metals related to health problems in the study area are recommended for future study.

Our study data indicate that some ASGM miners in the study area have experienced symptoms of chronic Hg intoxication, even though the Hg content of their hair did not demonstrate a harmful level. In other words, Hg level in the hair may be a weak biomarker of chronic Hg intoxication, especially in the case of elemental Hg exposure during ASGM processes. Instead, clinical examination is necessary for the assessment of chronic Hg intoxication in the ASGM community. A pulmonary function test can be used as a bioindicator tool. The estimation of urinary Hg should be considered for the indication of chronic Hg intoxication of the ASGM community, who are exposed to elemental Hg through inhalation. To obtain a better assessment of the health impact of ASGM on the community in Myanmar, we recommend that a health survey with a larger sample size should be conducted in the future.

## 5. Conclusions

Based on the findings of this preliminary study, some ASGM miners showed signs and symptoms of chronic Hg intoxication, including chronic tremor, numbing of the digits, and ataxic gait. The lung function was also found to be degraded, and the degree of degradation increased in correlation with the duration of mining. The concentration of Hg in miners’ hair is greater than that in non-miners’ hair, and Hg content in the hair of some of ASGM miners and non-miners was measured at the warning level. The non-miner group did not show abnormal clinical findings. Since this preliminary study has limitations, such as a small sample size and the variation in the characteristics of the participants, such as different age and BMI of the two groups, these findings alone cannot determine the health status of the studied community. However, as this was the first preliminary clinical survey conducted in the ASGM community in Myanmar, these findings are important as they demonstrate merit in support of future clinical studies of ASGM communities. We recommend that future studies conduct comparative health impact assessment surveys of the ASGM community in the study area with a larger sample size in the control area, and they should involve the analysis of other bioindicators such as a urinary Hg analysis, as an indicator of chronic Hg intoxication by elemental Hg, along with the medical indicators such as a pulmonary function test. 

## Figures and Tables

**Table 1:** Characteristics of the participants.

Characteristics	Miners	Non-Miners	*P* Value
Number of respondents	18	11	
Sex	12 male	6 male	0.51
	6 female	5 female	
Age, y (mean ± SD)	37.6 ± 15.2	56.1 ± 13.9	0.01
BMI (mean ± SD)	20.9 ± 4.6	25.4 ± 2.9	0.01

*P* values were calculated using the Mann–Whitney test, with *P* < 0.05 showing statistical significance.

**Table 2:** Analysis of heavy metals in hair samples.

Heavy Metals	Miners	Non-Miners
Hg, μg/g *	0.93 (0.72–1.44)	0.63 (0.53–0.67)
Pb, μg/g	6.09 (3.67–17.61)	5.26 (2.08–8.77)
As, μg/g	0.20 (0.12–0.33)	0.16 (0.11–0.24)
Cd, μg/g	0.04 (0.01–0.10)	0.05 (0.02–0.17)
Cu, μg/g	11.09 (10.11–13.27)	11.92 (10.82–15.09)

Values are expressed as median (interquartile range). * *P* = 0.01; Mann–Whitney test.

**Table 3:** Respiratory assessment including smoking status and parameters of spirometry test.

Respiratory Assessment	Miners	Non-Miners
Smoking status, *n*		
Smoker	8	2
Nonsmoker	10	9
Duration of smoking, y median (IQR)	18.5 (9.3–34.3)	39.5 (34.3–44.8)
No. of cigarettes smoked per day median (IQR)	4.0 (1.6–5.0)	1.5 (1.3–1.8)
Brinkman index median (IQR)	82.0 (6.5–171.3)	54.0 (52.0–56.0)
FVC, L median (IQR)	2.89 (2.26–3.37)	2.11 (1.77–2.59)
% Prediction of FVC median (IQR)	75.5 (64.5–86.8)	77.0 (55.5–80.0)
FEV1, L median (IQR)	2.49 (2.06–3.35)	2.01 (1.71–2.51)
% Prediction of FEV1 median (IQR)	81.5 (71.3–90.8)	83.0 (64.5–91.5)
Spirometry interpretation, *n* (%)		
Normal	7 (38.9%)	3 (27.3%)
Mild	5 (27.8%)	3 (27.3%)
Moderate/moderately severe	5 (27.8%)	5 (45.5%)
Severe	1 (5.6%)	-

Liter (L). Interquartile range (IQR).

**Table 4:** Comparison of the variables between miners and non-miners.

Variables				
	FVC	FEV1	Hg level of scalp hair	Pb level of scalp hair
*P* value	0.05	0.07	0.01	0.58

*P* < 0.05 was considered statistically significant; Mann–Whitney test.

**Table 5:** Correlation among the variables in miners and non-miners.

Classification	Miners	Non-miners
FVC and age	0.001 ^Δ^	0.001 ^Δ^
FEV1 and age	0.001 ^Δ^	0.001 ^Δ^
FVC and smoking	0.26 ^Δ^	0.01 ^Δ^
FEV1 and smoking	0.004 ^Δ^	0.01 ^Δ^
FVC and mining duration	0.001 ∗	-
FEV1 and mining duration	0.007 ∗	-
FVC and Hg level	0.68 ∗	0.80 ∗
FEV1 and Hg level	0.74 ∗	0.80 ∗
FVC and Pb level	0.12 ∗	0.70 ∗
FEV1 and Pb level	0.06 ∗	0.70 ∗

Data are presented as *P* values, with *P* < 0.05 showing statistical significance. ^Δ^ Mann–Whitney test. ∗ Spearman’s Rho correlation test.
